# Effect of Hancornia speciosa aqueous extract treatment on biochemical parameters in diabetic pregnant rats

**DOI:** 10.1186/1758-5996-7-S1-A76

**Published:** 2015-11-11

**Authors:** Rafaianne Queiroz de Moraes Souza, Leila Santos Neto, Daniela Gomes Alves, Thaigra de Sousa Soares, Kleber Eduardo de Campos, Madilene Francely Américo, Débora Cristina Damasceno, Gustavo Tadeu Volpato

**Affiliations:** 1 Universidade Federal de Mato Grosso, Barra do Garças, Brazil

## Background

Hancornia speciosa, commonly known as mangaba, is used by pregnant women for diabetes treatment, but their effects and possible maternal biochemical repercussions are unknown.

## Objective

To evaluate the effect of Hancornia speciosa aqueous extract treatment on biochemical parameters in serum and oxidative stress in liver of diabetic and non-diabetic pregnant rats.

## Materials and methods

Diabetes was induced by streptozotocin (40 mg/Kg) in virgin female Wistar rats. After diabetes induction, rats were mated. The pregnant diabetic rats were put in four experimental groups, with n minimum=12 animals/group: Non-diabetic; Non-diabetic Treated; Diabetic and Diabetic Treated. Hancornia speciosa leaf aqueous extract (600 mg/kg) was daily administered in all gestational period. On days 0, 7, 14 and 21 the glycemia were measured. On day 21 of pregnancy, all rats were anesthetized and killed. The blood and liver were collected. The biochemical parameters were analyzed for blood (glucose, alanine aminotransferase [ALT], total protein, total cholesterol, triglycerides, High Density Level [HDL]-cholesterol) and also in liver (malondialdehyde [MDA], superoxide dismutase [SOD], catalase, total glutathione, thiol group) for oxidative stress. Analysis of variance followed by Tukey's test were used. Differences were considered statistically significant when p<0.05.

## Results

After treatment with H. speciosa extract, non-diabetic and diabetic rats presented no glycemic changes. Therefore, all the experimental groups showed increasing in glutathione levels compared to control group. Both diabetic groups presented higher levels of triglycerides and cholesterol and ALT actives, and also decreasing in serum protein levels compared to non-diabetic animals. Moreover, the treatment with H. speciosa in diabetic group increased HDL-cholesterol and decreased malondialdehyde (MDA) levels compared to diabetic group.

## Conclusion

The aqueous extract from H. speciosa leaves failed to modify the maternal hyperglycemia, biochemical parameters and stress oxidative.

